# FcRn antagonist and C5 complement inhibitor as early rescue strategies in severe Myasthenia Gravis: a two-case report

**DOI:** 10.3389/fimmu.2026.1818496

**Published:** 2026-06-19

**Authors:** Mosè Parisi, Nicola Molitierno, Claudia Alberti, Delia Gagliardi, Daniele Velardo, Giacomo Pietro Comi, Stefania Paola Corti

**Affiliations:** 1 Department of Pathophysiology and Transplantation, University of Milan, Milan, Italy; 2 Neurology Unit, Department of Neuroscience and Mental Health, Fondazione Istituto di Ricovero e Cura a Carattere Scientifico (IRCCS) Ca’ Granda Ospedale Maggiore Policlinico, Dino Ferrari Centre, Milan, Italy; 3 Neuromuscular and Rare Diseases Unit, Department of Neuroscience and Mental Health, Fondazione Istituto di Ricovero e Cura a Carattere Scientifico (IRCCS) Ca’ Granda Ospedale Maggiore Policlinico, Dino Ferrari Centre, Milan, Italy

**Keywords:** efgartigimod, Myasthenia Gravis, neuroimmunology, ravulizumab, rescue therapy, thymectomy

## Abstract

**Background:**

Myasthenia gravis (MG) is an autoimmune neuromuscular disorder in which approximately 10–15% of patients with generalized AChR antibody–positive MG develop refractoriness to standard immunosuppressive therapies. Advanced therapeutic strategies, including FcRn antagonists and C5 complement inhibitors, have demonstrated early and sustained clinical efficacy in pivotal phase 3 trials. However, evidence supporting their early use in complex clinical scenarios remains limited.

**Case report:**

We report two cases of severe generalized MG in which early initiation of advanced therapies was associated with rapid clinical stabilization. In the first case, a 75-year-old man with thymoma-associated MG and severe bulbar involvement refractory (MG-ADL: 11) to plasma exchange (PLEX) and intravenous immunoglobulins (IVIg), and unable to continue azathioprine due to adverse events, received off-label efgartigimod preoperatively. Near-complete resolution of bulbar symptoms was observed within 48 hours, enabling robot-assisted thymectomy on day 4 following the first infusion, with sustained neurological improvement at one-month follow-up (MG-ADL score: 2). In the second case, a 74-year-old man with severe refractory bulbar MG requiring nasogastric tube feeding (MG-ADL: 13) and subsequent percutaneous endoscopic gastrostomy (PEG) was treated with ravulizumab after an incomplete response to PLEX, IVIg, corticosteroids, and azathioprine. MG-ADL decreased from 9 at treatment initiation to 5 after two infusions of ravulizumab, and complete recovery of swallowing function allowed PEG removal at 18-week follow-up, with achievement of minimal symptom expression (MG-ADL score: 0).

**Discussion:**

These cases highlight the potential role of early and targeted use of advanced immunotherapies in severe, refractory MG, including as a bridging strategy to thymectomy. Further prospective studies are needed to define optimal criteria and timing for early integration of these agents into the therapeutic algorithm.

## 1 Introduction

Myasthenia gravis (MG) is an autoimmune disorder of the neuromuscular junction, characterized in approximately 80% of cases by the presence of antibodies directed against the acetylcholine receptor (AChR) (1). As an autoimmune disease, MG is primarily treated with immunosuppressive and immunomodulatory therapies, including corticosteroids, azathioprine, mycophenolate mofetil, intravenous immunoglobulins (IVIg), and plasma exchange, the latter most commonly employed as rescue therapy (2). Despite these treatment options, approximately 10–15% of patients with generalized AChR antibody–positive MG develop refractoriness to standard therapies (3, 4). Over the past decades, this unmet clinical need has driven the development of advanced therapeutic strategies with novel mechanisms of action. These include FcRn antagonists, which inhibit immunoglobulin G (IgG) recycling - thereby reducing circulating autoantibodies - through targeting of the neonatal Fc receptor (FcRn) (5, 6), and complement inhibitors targeting the C5 level, aimed at preventing membrane attack complex (MAC) formation and subsequent postsynaptic membrane damage (7–10). Furthermore, clinical trials have demonstrated that both agents provide an early and sustained clinical response, with improvements in Myasthenia Gravis Activities of Daily Living (MG-ADL) (11) and Quantitative Myasthenia Gravis (QMG) scores (12, 13), supporting their potential use as early or rescue therapy, particularly for stabilization of patients with thymoma-associated myasthenia gravis during the perioperative period (14–16). We present two instructive cases that illustrate the potential of advanced therapies to influence the disease trajectory in challenging clinical settings, offering insights that may inform therapeutic decision-making in similar situations.

## 2 Case presentation

### 2.1 Clinical case 1

A 75-year-old man with a history of arterial hypertension, radiotherapy-treated prostate cancer (currently in remission), and past hepatitis B infection presented with a subacute onset of generalized asthenia, subsequently developing bulbar symptoms including dysarthria with nasal speech and mild dysphagia. Four months later, he experienced a self-limited episode of dyspnea, prompting cardiological evaluation that was unremarkable, and chest computed tomography (CT) revealed a para-aortic mediastinal mass with calcified walls of uncertain etiology.

However, within one month, the patient’s neurological condition had significantly deteriorated and neurological examination on admission revealed severe fatigability, marked dysarthria and hypophonia, nasal speech, bilateral ptosis, facial weakness, and severe dysphagia requiring nasogastric tube feeding (MG-ADL: 11). Repetitive nerve stimulation confirmed a postsynaptic neuromuscular junction disorder, and serum anti-AChR antibodies were detected (22.6 pmol/mL), establishing a diagnosis of generalized MG.

Pyridostigmine was started and titrated up to 45 mg four times daily. Initial thoracic CT scans were inconclusive for thymoma; however, contrast-enhanced chest magnetic resonance imaging (MRI) identified a non-invasive cystic thymoma (5.5 × 3.5 cm) with both solid enhancing and cystic components. Upon admission (QMG: 12), the patient underwent five sessions of plasma exchange (PLEX) administered on alternate days. Prednisone was initiated at 10 mg/day, 7 days after the start of PLEX, and progressively titrated up to 1 mg/kg/day to minimize the risk of steroid-induced initial worsening.

Due to incomplete clinical response to PLEX, IVIg therapy was initiated immediately after completion of the PLEX cycle (2 g/kg over five days) and 3 days after prednisone initiation. Azathioprine was initiated but discontinued due to the concurrent development of mild normocytic anemia, severe thrombocytopenia, and femoral vein catheter-related thrombosis.

Given the persistence of fluctuating bulbar symptoms and the onset of proximal weakness (MG-ADL score ranging between 10 and 12), and in view of impending thymectomy, off-label efgartigimod (10 mg/kg) was administered preoperatively, approximately one month after completion of IVIg therapy. Rapid and marked clinical improvement was observed within 48 hours, with near-complete resolution of bulbar symptoms - persistent mild rhinolalia only after prolonged speech - and minimal ptosis, along with a significant reduction in MG-ADL score to 4 (QMG: 4). Robot-assisted left radical thymectomy was performed four days after the first efgartigimod infusion. Histopathology revealed a WHO type A thymoma (pT1a, Masaoka–Koga stage IIb).

Postoperative efgartigimod infusions were continued for a total of four weekly administrations, resulting in sustained neurological improvement. At one-month follow-up, the patient remained clinically stable with minimal residual ptosis (Myasthenia Gravis Foundation of America (MGFA) class I, MG-ADL score 2, QMG: 2), without dysphagia, respiratory compromise, or limb weakness.

### 2.2 Clinical case 2

A 74-year-old man with an unremarkable past medical history was referred to our center for progressively worsening ocular and bulbar symptoms that had developed over the preceding four weeks. He had initially been evaluated at another institution for the onset of fluctuating right-sided ptosis and diplopia. MG was suspected, and serum anti-AChR antibodies were detected (4 pmol/mL). Repetitive nerve stimulation electromyography confirmed a postsynaptic neuromuscular junction disorder.

Shortly after its introduction, pyridostigmine was discontinued due to adverse effects, followed by worsening bulbar symptoms, including dysphagia, hypophonia with rhinolalia, and bilateral ptosis.

On admission, neurological examination revealed hypophonic, dysarthric speech with rhinolalia. Severe left-sided ptosis was present, with near-complete eyelid closure, and fatigable ptosis of the right eye after sustained upward gaze. Fatigability of the orbicularis oculi muscles was evident, while the strength of the orbicularis oris, buccinator, and masseter muscles was preserved. Mild tongue weakness and a weak cough were noted. Limb strength was normal in all four extremities.

Given the severity of dysphagia for both solids and liquids, a nasogastric tube was placed (MG-ADL score: 13; QMG: 12; MGFA class IVb). Contrast-enhanced chest CT showed no evidence of thymic hyperplasia or thymoma. Pyridostigmine was reintroduced, resulting in minimal improvement of ocular symptoms but recurrence of dose-limiting adverse effects, necessitating dose optimization.

Due to persistent and severe bulbar involvement, the patient underwent therapeutic PLEX (four sessions on alternate days). Prednisone was started after the first PLEX session and titrated up to 1 mg/kg/day with a step-up regimen. IVIg therapy (2 g/kg over 5 days) was initiated immediately after completion of the PLEX cycle, and azathioprine was introduced on day 3 of the IVIg course, with titration up to 2 mg/kg/day. Although gradual improvement in diplopia, ptosis, and hypophonia was observed, dysphagia remained severe and disabling despite conventional immunosuppressive treatment (MG-ADL: 9). Twenty days after completion of the IVIg cycle, following microbiological screening and prophylactic antibiotic therapy, treatment with the C5 complement inhibitor ravulizumab was initiated at a weight-based loading dose of 2400 mg intravenously, followed by a maintenance dose of 3000 mg two weeks later. At ravulizumab initiation, MG-ADL had improved from 13 to 9 following rescue therapies (QMG: 7). Following ravulizumab initiation, further clinical improvement was observed, with the MG-ADL score decreasing to 5 at discharge (QMG: 3) after completion of two infusions. Nevertheless, severe dysphagia remained the predominant refractory manifestation, requiring percutaneous endoscopic gastrostomy (PEG) placement 6 weeks after ravulizumab initiation. Notably, the most relevant change concerned bulbar function, with progressive recovery of swallowing capacity over the subsequent weeks. At the 18-week outpatient follow-up (three administrations completed), complete recovery of swallowing function allowed PEG removal, with achievement of minimal symptom expression (MSE, MG-ADL score: 0, QMG: 0).

**Figure 1:**
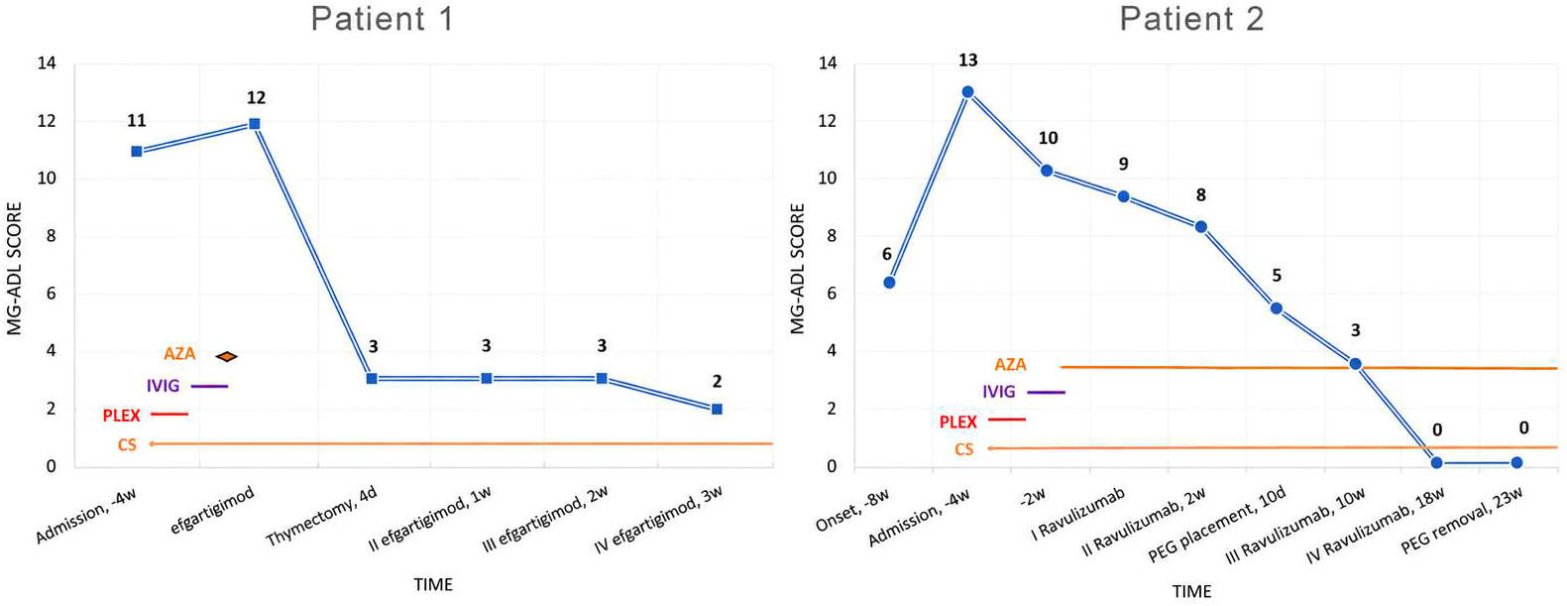
Longitudinal MG-ADL changes in Patient 1 and Patient 2. Major therapeutic interventions (PLEX, IVIg, AZA: azathioprine, CS, corticosteroids, efgartigimod, ravulizumab), thymectomy (Case 1), and PEG placement/removal (Case 2) are indicated. W = weeks, d, days.

Longitudinal changes in MG-ADL scores over time in the two reported cases are shown in **Figure 1**.

## 3 Discussion

The approval of advanced therapies has significantly improved the management of complex patients who were previously reliant on repeated rescue treatments, frequent hospitalizations, and prolonged corticosteroid exposure associated with substantial adverse effects. Notably, these therapies enable clinical stabilization with a marked steroid-sparing effect. However, in many European healthcare systems, access to advanced therapies is often contingent upon documentation of inadequate response to conventional treatments administered at appropriate dosages and durations. This requirement, combined with the delayed onset of clinical efficacy of immunosuppressive agents, represents a major limitation for clinicians, particularly in patients with severe or complicated disease onset who may be refractory to first-line therapies (17). Consequently, clinicians often face prolonged periods before being able to consider first-line immunosuppressive therapy as unsuccessful, with a significant impact on patients’ quality of life, frequent hospitalizations, and the use of limited resources such as IVIg. An additional layer of complexity is introduced by the presence of a thymic mass, reported in approximately 15% of patients with generalized MG (18, 19), which can complicate symptom management and hinder disease stabilization, making it more challenging to optimize the patient’s condition prior to thymectomy and to perform surgery as early and safely as possible (19). Moreover, pivotal phase 3 clinical trials of these two agents - ADAPT (12) for efgartigimod and CHAMPION-MG (13) for ravulizumab - have demonstrated significant clinical improvement within the first two weeks of treatment initiation, with an increasing number of reports describing early administration to achieve prompt clinical stabilization. This strategy may also facilitate timely surgical intervention, allowing patients to undergo thymectomy under more stable clinical conditions, as reported in a phase II Chinese clinical trial (16) assessing the safety and efficacy of perioperative efgartigimod use. Against this background, we report two clinical cases illustrating the potential impact of early use of advanced therapies. The first case is emblematic of early intervention with efgartigimod, leading to rapid clinical stabilization in a patient with a highly unstable presentation characterized by bulbar symptoms, thereby allowing timely advancement to thymectomy. Wang et al. (16), in their phase II study, proposed a “2 + 2 regimen” consisting of two preoperative efgartigimod infusions, thymectomy, and two subsequent postoperative infusions, resulting in rapid and safe improvement of neuromuscular symptoms during the perioperative period. In our patient, however, thymectomy was performed on day 4 following the first infusion, while maintaining optimal disease control, reflected by an MG-ADL score of 2, until the end of the treatment cycle. To date, evidence on the optimal timing and dosing of efgartigimod in the perioperative setting remains limited (14, 15, 20, 21), and no established protocols have been defined, underscoring the need for prospective studies in this specific clinical context. Furthermore, recent evidence supports the rapid onset of action of efgartigimod even when used as add-on therapy in myasthenic crisis, with a favorable safety profile (22). The rapid perioperative stabilization observed in our patient is consistent with the immunological mechanism of FcRn inhibition (14). By preventing endosomal recycling of IgG, FcRn blockade accelerates IgG degradation, leading to a rapid, reversible reduction in circulating pathogenic anti-AChR antibodies. In this sense, efgartigimod may act as a form of “molecular apheresis,” enabling prompt disease control while bridging the patient to the delayed maximal efficacy of conventional immunosuppressive therapy (5, 6, 23). Finally, this case highlights the importance of choosing the appropriate imaging modality for thymoma detection, particularly in poorly controlled MG. As suggested by published case reports, chest MRI is more sensitive than CT for identifying cystic thymomas, owing to its superior characterization of fluid components (24, 25). The second case demonstrates that early ravulizumab initiation in a newly diagnosed patient with severe, refractory bulbar MG may enable rapid clinical stabilization and prompt achievement of MSE, including PEG removal. Recovery of swallowing function became evident soon after treatment initiation and occurred well ahead of what would typically be expected given the delayed onset of action of conventional immunosuppressive therapies. This clinical response is mechanistically consistent with C5 complement inhibition. By blocking cleavage of C5 and preventing formation of the MAC, ravulizumab interrupts complement-mediated postsynaptic damage at the neuromuscular junction (26–28). These findings support the hypothesis that, in patients with persistent bulbar dysfunction, early complement blockade may attenuate ongoing complement-mediated postsynaptic injury, thereby promoting functional recovery of swallowing, although the gradual improvement observed in this case may partly reflect both the pharmacodynamic profile of C5 inhibition and the time required for structural and functional remodeling of the neuromuscular junction. However, this interpretation should be made with caution, as dysphagia is a complex symptom influenced not only by neuromuscular junction dysfunction but also by the coordinated interaction of multiple muscle groups, mucosal structures, and other swallowing-related mechanisms. As a result, recovery of dysphagia may be more prolonged and less predictable than that observed for ocular symptoms. In conclusion, this case series supports the potential benefit of early and targeted access to advanced therapies in patients presenting with complex disease courses from onset, in whom rescue therapies fail to achieve adequate clinical stabilization. Early initiation of these agents may promote rapid symptom control and, in selected cases, serve as a bridging strategy to standard immunosuppressive therapy until its maximal efficacy is achieved. Further studies are needed to define optimal criteria for early initiation of advanced therapies and to assess their long-term impact on disease outcomes and healthcare resource utilization.

## Data Availability

The original contributions presented in the study are included in the article/supplementary material. Further inquiries can be directed to the corresponding author.
